# Clinical Risk Score for Predicting Vascular Dementia after Ischemic Stroke in Thailand

**DOI:** 10.1155/2022/1600444

**Published:** 2022-09-26

**Authors:** Pornpatr A. Dharmasaroja, Thammanard Charernboon

**Affiliations:** ^1^Department of Medicine, Faculty of Medicine, Thammasat University, Pathumthani, Thailand; ^2^Department of Psychiatry, Faculty of Medicine, Thammasat University, Pathumthani, Thailand; ^3^Center of Excellence in Applied Epidemiology, Thammasat University, Pathumthani, Thailand

## Abstract

**Background:**

Poststroke dementia is an important consequence of stroke and warrants early prevention, detection, and management. The objective of the study was to develop a simple clinical risk score for predicting risk of vascular dementia in patients with ischemic stroke.

**Methods:**

The design was a prospective cohort study with 177 ischemic stroke survivors. A standard stroke evaluation was performed at admission, and dementia evaluation was conducted at six months after stroke. The significant predictors were used to develop a risk score using a multivariable logistic regression model.

**Results:**

Six months after stroke, 27.1% of the patients were diagnosed with vascular dementia. Five predictors were used in the risk score: age, education, history of stroke, white matter hyperintensities, and stroke subtype. The risk score had an area under receiver operating characteristic curve (AuROC) of 0.76, 72.9% sensitivity, and 79.1% specificity in predicting risk of vascular dementia. The predicted probability of vascular dementia for each risk score point was also reported.

**Conclusion:**

The clinical risk score had an acceptable accuracy in predicting vascular dementia in ischemic stroke survivors. It can be used for identifying those who are at a high risk of developing vascular dementia.

## 1. Introduction

Stroke is one of the major causes of disability worldwide [1]. Besides its effect on physical disability, cognitive impairments after stroke can also cause a disability and prevent the stroke survivors from living independently. Previous studies show that at 3-6 months poststroke, the prevalence of vascular dementia was 10-20% [2–4].

Existing literature suggests that some factors are predictors of cognitive impairment after strokes such as older age, low level of education, diabetes, atrial fibrillation, recurrent stroke, stroke severity, and white matter lesions [2, 3, 5]. Among the many risk factors, organizing them in a structured manner and easy to use risk score would make them more useful for clinicians to monitor and identify stroke survivors at risk for vascular dementia. It could also be useful for precision medicine, i.e., selecting high-risk patients into clinical trials aiming to reduce the conversion rate of dementia.

There are two recently developed risk scores in stroke survivors, i.e., SIGNAL and CHANGE scales [6, 7]. Both of them were designed to identify poststroke cognitive impairment which has varying degrees of impairment from mild cognitive impairment to dementia. However, in this study, we aim to identify only vascular dementia since these patients have clear disability, impacts on caregivers, and need continuing care. Moreover, the SIGNAL study included magnetic resonance angiography (MRA) variables that are not available for every patient in many stroke units in developing countries. The objective of this study was to develop a simple clinical risk score that can identify stroke survivors who are at risk for developing vascular dementia within 6 months poststroke.

## 2. Materials and Methods

### 2.1. Participants

The study design was a prospective cohort study. The participants were patients with acute ischemic stroke admitted at Thammasat University Hospital during June 2018 to April 2019. Diagnosis of stroke was made by stroke neurologists based on clinical symptoms, and a new stroke lesion was confirmed by the finding of magnetic resonance imaging (MRI). Upon discharge, participants were scheduled for outpatient follow-up at 1, 3, and 6 months later.

The exclusion criteria were those with (1) a history of dementia prior to stroke onset, (2) aphasia, (3) major psychiatric illness (i.e., schizophrenia or bipolar disorder), (4) recurrent strokes during the six-month follow-up period, (5) presentation to the outpatient clinic outside 5–7 months, and (6) any severe medical condition disease (e.g., decompensated cirrhosis) or severe physical/neurological disability that precluded cognitive assessment.

All included patients received magnetic resonance imaging. Ischemic stroke subtypes were classified by the Trial of Org 10172 in Acute Stroke Treatment (TOAST) [8]. It consists of five subtypes: large-artery atherosclerosis, cardioembolism, small-vessel occlusion, stroke of other determined etiology, and stroke of undetermined etiology. White matter hyperintensity lesion was classified using the Fazekas scale. Stroke severity was assessed by the National Institutes of Health Stroke Scale (NIHSS). The study was approved by the Human Ethics Committee of Thammasat University (protocol number MTU-EC-IM-2-206/60).

### 2.2. Diagnosis of Dementia

Clinical diagnosis of vascular dementia was made by senior neurologists at 6 (±1) months after the stroke based on NINDS–AIREN criteria [9]. Cognitive function was assessed using the Montreal Cognitive Assessment and Thai Mental State Examination [10, 11]. Vascular dementia was diagnosed if the patients had cognitive impairment in two or more cognitive domains and caused deficits in activities of daily living. Mild cognitive impairment (MCI) was diagnosed if the patients had cognitive impairment, but it did not cause deficits in activities of daily living [12]. Patients with normal cognitive function were classified as cognitively normal controls. Since this study's aim was to develop a risk score for predicting only vascular dementia, therefore, the MCI and cognitively normal control groups were combined together as a nondementia group.

### 2.3. Candidate Predictors

We collected a wide range of predictors based on existing previous studies including characteristics (gender, age, and level of education), underlying diseases (diabetes mellitus, hypertension, hyperlipidemia, coronary artery disease, and atrial fibrillation), subtype of stroke, severity of stroke, white matter hyperintensities, and cerebral microbleeds. These variables were agreed by the research team that they were common and available in real-life clinical practice to ensure that the risk score can be practically implemented in routine clinical stroke investigations.

### 2.4. Development of Clinical Risk Score and Statistical Analysis

Potential predictors for the risk score were firstly identified by comparing patients with dementia and patients without dementia at 6 months and tested for statistical significance using exact test or independent *t*-test. Predictors with *p* value < 0.2 at a univariable analysis were put into stepwise backward logistic regression models with dementia status as the outcome variable. Variables with *p* value ≥ 0.2 were removed from the model. In this multivariable analysis, continuous and categorical variables were grouped into binary variables to create a parsimonious model and simple to use scoring system. The point system for the risk score was developed based on the coefficients from the final logistic model.

Predictive accuracy was assessed using area under receiver operating characteristic curve (AuROC), sensitivity, specificity, positive predictive value, negative predictive value, and likelihood ratio if test positive. The cutoff point of the risk score was identified based on a balance between sensitivity and specificity. Calibration was examined by plotting predicted probability of the risk score against the actual probability of the patients who developed vascular dementia at every risk score point. All analyses were performed using STATA statistical software version 14.0 (StataCorp LP, TX, USA). The dataset had no missing values.

## 3. Results

Among the total of 177 patients in the cohort, 48 (27.1%) were diagnosed with vascular dementia at about six months after the stroke. The remaining 89 (50.3%) were diagnosed with mild cognitive impairment, and 40 (22.6%) were cognitively normal participants.

Table 1 shows the demographics, clinical factors, and univariable analysis between patients with vascular dementia and patients who did not have a diagnosis of dementia. Patients with vascular dementia had lower educational levels, higher age, and history of stroke. Among the stroke-related predictors, participants with large-artery atherosclerosis/cardioembolism and white matter hyperintense lesions were more likely to have vascular dementia.

### 3.1. Risk Score Development

Six predictors with *p* value < 0.2 from univariable analysis (age, education, atrial fibrillation, history of stroke, stroke subtypes, and white matter lesions) were included into stepwise backward logistic regression models with dementia status as the outcome variable. The final regression model consisted of five predictors: age, education, history of stroke, white matter hyperintensities, and stroke subtype (Table 2). Among these variables, age (≥70 years) and educational level (≤6 years) seem to be the strongest predictor with the odds ratio of 8.1 and 4.0, respectively. The AuROC of the final regression model was 0.85.

The item score for each variable was assigned by calculating its regression coefficients divided by the lowest coefficient value (0.6) of the model and rounded to the nearest integer. A summary risk score was obtained by adding up all five item scores (Tables 2 and 3). For example, a 75-year-old patient with 4-year education experiencing first stroke due to large artery atherosclerosis without white matter hyperintense lesions, the total risk score is 4 + 2 + 1 = 7 points.

### 3.2. Validation and Calibration

The risk score has a score range from 0 to 11 points. A score of 5 or higher was identified as the optimal cutoff point with the AuROC of 0.76, sensitivity of 72.9%, and specificity of 79.1% (Table 4). We also calculated the predicted probabilities for each risk score point in Table 5. At 0 point, the probability of vascular dementia is 3.2%, while at 11 point, the risk is 92.3%.

**Figure 1:**
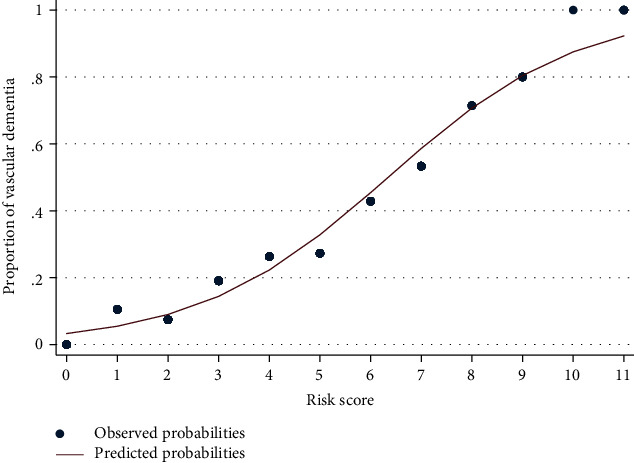
Calibration graphs plotting the predicted probabilities of the risk score (solid line) against the observed probabilities (scatter plot) of vascular dementia.

Predicted probabilities of the risk score were plotted against actual proportions of the vascular dementia at every risk score point (Figure 1). The risk score showed good calibration since it illustrated that the predicted probability of the risk score closely followed the actual trends of the prevalence of vascular dementia.

## 4. Discussion

The study demonstrated that our simple 11-point risk score was sensitive and valid in predicting ischemic stroke patients for significant risk of developing vascular dementia at six months poststroke.

The predictors in our risk score are consistent with previous studies as being important risk factors for vascular dementia. Age, education, white matter change, and recurrent strokes are common risk factors that were usually reported by the existing literatures [2, 3, 13]. In our risk score, age was the highest risk factor and had the highest point (4 points). Therefore, for stroke patients with age ≥ 70 years, the interpretation is simpler. If they have other risk factors, they are at high risk for developing vascular dementia.

Our risk score would be beneficial in screening stroke survivors at high risk for vascular dementia at six months. At-risk patients might then be prioritized for close regular follow-up; and preventive/rehabilitation interventions, such as medication or intensive rehabilitation programs, might be initiated at appropriate time. A risk score is also of value in clinical trials. Although trials for preventing post stroke cognitive impairment have not been promising [14, 15], there are some important limitations, such as the participants being heterogeneous and having a varied risk profile. That is, in terms of precision medicine, the risk score could be used as a screening tool to recruit only patients with high risk for dementia for these clinical trials.

One advantage of our risk score is that we provided both an optimal cutoff score as a simple way to identify patients with high risk, and the probability of developing vascular dementia for each risk score point. Using cutoff score ≥ 5, our model demonstrates acceptable sensitivity (72.9%), specificity (79.1%), and good NPV (88.7%). Thus, the risk score might also be useful in identifying stroke survivors who are unlikely to develop vascular dementia. Alternatively, clinician can determine patients' risk based on the risk score obtained (Table 5), such as patients with a risk score of 2 points or lower has a probability of developing vascular dementia of less than 9%, while a score of 9 points or higher, the risk for vascular dementia is higher than 80%. Clinicians can use these probabilities to consider further management for their patients.

Comparing two recently developed risk models in stroke patients, SIGNAL and CHANGE scores, our score demonstrated comparable performance with these two scoring systems (the study AuROC 0.85, CHANGE AuROC 0.74-0.82, and SIGNAL AuROC 0.78-0.83) [6, 7]. However, the main difference is that both SIGNAL and CHANGE scores are aimed at identifying poststroke cognitive impairment, not dementia.

### 4.1. Limitation

Patients with severe stroke or aphasia were excluded because we could not assess their cognitive function. This would underestimate the prevalence of the vascular dementia and the risk score could not be applied to these patients. As the participants in this study were Asian, the generalizability of the risk score to non-Asian population might not be applicable and would need further studies. Another limitation is that our cohort included only patients who had undergone MRI; therefore, it could not be used in stroke units with only CT available. External validation is also needed to validate the risk score in varied stroke settings.

## 5. Conclusions

In conclusion, the study was the first clinical risk score in identifying ischemic stroke patients for risk of developing vascular dementia in Thailand. The risk score demonstrated good performance in detecting 6-month onset vascular dementia. It is simple, easy to use, and applicable at bedside.

## Figures and Tables

**Table 1:** Characteristics and univariable analysis of patients with vascular dementia vs. nondementia patients.

Variables	Nondementia (*n* = 129)	Vascular dementia (*n* = 48)	*p* value
Gender: male, *n* (%)	89 (69.0%)	29 (60.4%)	0.288
Age (years): mean (SD)	61.7 (11.2)	74.5 (9.6)	<0.001
Education (years): mean (SD)	9.4 (5.0)	6.2 (4.3)	0.001
Hypertension, *n* (%)	94 (72.9%)	38 (79.2%)	0.443
Diabetes mellitus, *n* (%)	48 (37.2%)	15 (31.3%)	0.486
Hyperlipidemia, *n* (%)	105 (81.4%)	41 (85.4%)	0.658
Coronary artery disease, *n* (%)	7 (5.4%)	5 (10.4%)	0.311
Atrial fibrillation, *n* (%)	11 (8.5%)	9 (18.8%)	0.065
History of stroke, *n* (%)	12 (9.3%)	12 (25%)	0.012
NIHSS: mean (SD)	4.7 (4.0)	4.4 (3.3)	0.749
Stroke subtypes, *n* (%)			0.046
Small-artery occlusion	44 (34.1%)	11 (22.9%)	
Cardioembolism	12 (9.3%)	11 (22.9%)	
Large-artery atherosclerosis	39 (30.2%)	18 (37.5%)	
Undetermined cause and others	34 (26.4%)	8 (16.7%)	
White matter hyperintense lesions (Fazekas scale), *n* (%)			0.001
0	21 (16.3%)	4 (8.3%)	
1	79 (61.2%)	18 (37.5%)	
2	22 (17.1%)	16 (33.4%)	
3	7 (5.4%)	10 (20.8%)	
Cerebral microbleeds, *n* (%)			0.892
0	85 (71.4%)	30 (69.8%)	
1-3	30 (25.2%)	11 (25.6%)	
4-10	4 (3.4%)	2 (4.6%)	

NIHSS: National Institutes of Health Stroke Scale.

**Table 2:** Final multivariable logistic regression model with vascular dementia as the outcome variable.

Predictor variables	Coefficient	Odds ratio (95% CI)	*p* value	Score
Age				
<70	Ref			+0
≥70	2.1	8.1 (3.4, 19.1)	<0.001	+4
Education				
>6	Ref			+0
0-6	1.4	4.0 (1.7, 9.4)	0.001	+2
History of stroke				
No	Ref			+0
Yes	1.2	3.2 (0.9, 10.7)	0.054	+2
White matter hyperintense lesions (Fazekas scale)				
0-1	Ref			+0
2-3	0.9	2.4 (1.0, 5.8)	0.05	+2
Stroke subtype				
Others	Ref			+0
Large artery atherosclerosis or cardioembolism	0.6	1.8 (0.8, 4.3)	0.153	+1

Pseudo *R*^2^: 0.304; AuROC: 0.84.

**Table 3:** Clinical risk score for vascular dementia after ischemic stroke (HASTE).

Clinical risk score for vascular dementia after ischemic stroke (HASTE)		
Hyperintensities in white matter regions (Fazekas scale)	0-1	+0
	2-3	+2
Age	Below 70 years	+0
	70 years and above	+4
Stroke history	No	+0
	Yes	+2
Type of stroke	Others	0
	Large artery atherosclerosis or cardioembolism	+1
Educational level	7 years and above	+0
	Less than 7 years	+2
Score ranges 0-11 points		Total =
Score of 5 or more indicates significant risk of vascular dementia		

**Table 4:** Accuracy of the risk score.

Cutoff point	AuROC (95% CI)	Sensitivity (95% CI)	Specificity (95% CI)	PPV (95% CI)	NPV (95% CI)	LR+ (95% CI)
≥5	0.76 (0.69, 0.83)	72.9% (58.2, 84.7)	79.1% (71.0, 85.7)	56.5% (43.3, 69.0)	88.7% (81.4, 93.8)	3.5 (2.4, 5.1)

AuROC: area under receiver operating characteristic curve; PPV: positive predictive value; NPV: negative predictive value; LR+: likelihood ratio if test positive.

**Table 5:** Predicted probabilities of vascular dementia for each risk score.

Risk score	Probability of vascular dementia (%)	Risk score	Probability of vascular dementia (%)
0	3.2%	6	45.4%
1	5.5%	7	58.6%
2	8.9%	8	70.7%
3	14.4%	9	80.4%
4	22.3%	10	87.5%
5	32.8%	11	92.3%

## Data Availability

The data used to support the findings of this study are available from the corresponding author upon request.
